# Short-Term Response of Soil Spiders to Cover-Crop Removal in an Organic Olive Orchard in a Mediterranean Setting

**DOI:** 10.1673/031.012.6101

**Published:** 2012-05-13

**Authors:** Manuel Cárdenas, Juan Castro, Mercedes Campos

**Affiliations:** ^1^ Department of Botany and Zoology, Masaryk University, Kotlá˘ská 2, 61 137, Brno, Czech Republic; ^2^ department of Environmental Protection, Estación Experimental del Zaidín (CSIC), Profesor Albareda n° 1, 18008, Granada, Spain; ^3^ IFAPA Centro Camino de Purchil, Junta de Andalucía, P.O. Box 2017, 18080, Granada, Spain

**Keywords:** Araneae, cover crops, disturbance, ground dwelling spiders, olive agroecosystem

## Abstract

This study shows that disturbance caused by cover-crop removal (CCR) in an organic olive orchard affects ground-spider populations. The effect of CCR on various organic olive-orchard plots was assessed by monitoring the abundance and diversity of ground-dwelling spiders. Covered plots in the organic olive orchard were compared with uncovered plots where the covers had been removed mechanically. CCR positively affected the most abundant spider species *Zodarion styliferum* (Simon) (Araneae: Zodariidae) as well as other species of running spiders belonging to the families Gnaphosidae and Lycosidae. Over time, the two types of plots did not significantly differ in diversity or dominance. Similarly, no differences were detected between the study plots in terms of the distribution of individuals when a cluster-similarity analysis was performed. This lack of difference in diversity might be due to the spatial scale used in the study or climatology. Because of their general effects, CCR profoundly changed the abundance of spiders in the olive orchard, but with no clear impact on spider diversity.

## Introduction

The olive-orchard agroecosystem represents one of the most important crops in southern Spain (Civantos 2004), and thus it is informative to study the impact that covercrop removal (CCR), a supplementary landmaintenance technique, exerts on the associated predator arthropods, particularly spiders (Cárdenas et al. 2006; Cotes et al. 2009). CCR is vigorous farm work that subjects the ground and its inhabitants to a major disturbance, and is a prevailing cultural practice in Spanish olive groves (Pastor 2004). Farmers clear the ground for both fire prevention and the positive aesthetic of clean olive fields. This cultural practice opposes the recommendations of Cross-Compliance (Common Agricultural Policy, UE for erosion control: conditionality). Vegetal cover on the ground is known to protect against direct scarring of raindrops and erosion. Aside from this disadvantage, this study investigated whether CCR affects the spider fauna.

Land-maintenance systems in organic olive orchards are based on the use of cover crops, which are mechanically controlled with mowers. The plant remains are either left on the surface or buried, thereby eliminating the cover crop (Guzmán and Alonso 2004). Nonorganic olive orchards also practice CCR at the end of spring, presumably because olive growers consider weeds to indicate neglect of orchard maintenance, and because they seek to maintain an easily accessible and regular ground surface to pass with the tine harrow (Pastor 2004); that is, one of the types of harrows (secondary tillage for seedbed preparation or light surface cultivation) with an operating depth about 1–2 inches, and operating speed from 4 to 7 miles per hour (NRCS 2005).

The control of surface erosion from water in olive orchards requires the constant presence of plant remains on the surface, mainly to protect the ground from the direct impact of raindrops (Castro 1993; De Luna 1994; Gómez et al. 2004). These complementary improvements in the soil's physical, chemical, and biological properties favor biodiversity, by increasing the content of organic matter in the surface layers (thereby increasing infiltration), reducing water loss through evaporation, and reducing lixiviation and nutrient leaching, thereby improving the water balance in olive orchards. Consequently, if olive orchards with crop covers are correctly managed, good harvests and long-term productivity are ensured (Castro et al. 2008). The tolerance of macroinvertebrate taxa to physicochemical stress has been widely used in the analysis and interpretation of field data (Carlisle et al. 2005). Several spider studies demonstrate that disturbances such as fire (Urones and Majadas 2002), flooding (Bonn et al. 2002), agricultural management systems (Haskins and Shaddy 1986; Cattin et al. 2003; Pfiffner and Luka 2003), and the use of pesticides (Kennedy et al. 2001; Bell et al. 2002) diminish spider abundance.

However, the effect that these disturbances may have on diversity will depend on the degree to which this disturbance affects the particular ecosystem. Consequently, very aggressive disturbances (i.e., fire) will decrease the diversity of predators such as spiders, ants, and beetles—at least initially (Oliver et al. 2000; Urones and Majadas 2002). Other moderate disturbances can augment species richness (Connell 1978), as for example the temporary presence of vegetation that could promote diversity, according to the hypothesis that intermediate disturbances provide greater species diversity than larger or smaller disturbances (Huston 1979, 1994; Aronson and Precht 1995). In the case of spiders, an exception has been found to this trend in areas with temporary vegetation such as coastal dunes (Bonte et al. 2004), due to the narrow range of the environmental variation studied. There are, however, examples of different taxa where this hypothesis of intermediate disturbance can be verified, such as in fouling communities (Lenz et al. 2004).

Spiders, an abundant and diverse group of arthropods (Turnbull 1973), constitute a group of predators that can be classified into various groups according to their hunting strategy. For example, Uetz et al. (1999) distinguishes two major guilds: active cursorial spiders and web-spinning spiders. These guilds, in turn, have other subgroups depending on their ecological strategy. The short-term effects (positive or negative) of the elimination of cover vegetation on the spider community in an organic olive grove were investigated, as well as how these effects developed throughout the first months, since this is one of the most widely used techniques among non-tillage cultural practices in olive orchards. In comparing disturbed and undisturbed olive groves, both the possible negative effects on abundance and diversity and the subsequent immigration from adjacent areas could be documented. The aim of this study was to determine the impact of tine harrowing CCR, and thus the existence of bare ground, on land arthropods, particularly ground-dwelling spiders.

## Materials and Methods

### Study site

The study was conducted in a large olivegrowing area near Mancha Real, Jaén, Spain, in an orchard called Peñaflor. The 25-ha orchard consists of single-trunk cv. Picual olive trees *Olea europa* L. (Lamiales: Oleaceae) with crown diameters ranging from 1.5 to 3 m, and annually increasing productivity currently between 20 and 30 kg/tree. The orchard was established in 1994 with an 8 × 8 m planting pattern (156 olive trees/ha). The orchard has an underground irrigation system and the olives are mechanically harvested. Olive-orchard maintenance was conventional until 2002, and since 2005 the farm has been organically certified. This organic farm was chosen in order to eliminate the possible effects of pesticides on the arthropod fauna.

### Experimental design

The land-maintenance technique used in the experiment was that of weed cover crops controlled by constant mowing. The mower blade repeatedly strikes the ground, leveling the soil and eliminating unevenness. The mowing frequency depended on the plantcover development, and normally the species completed their cycle and produced seeds. Cover crops present in the plot were entirely dominated by native spontaneous grasses, with clumps of *Bromus* sp., *Hordeum* sp., and *Diplotaxis* sp. All the species were annuals. During the study year, the cover was not particularly well developed, and the cover species were Gramineae.

Pre-sample intervention (CCR) consisted of passing the tine harrow over the dry debris of the plant cover cut by the mower. The tine harrow broke up the surface crust and broke apart the remains of the existing cover, mixing them in the first few millimeters of the soil. The debris and seeds remained on the soil or were partially buried. The harrow passed four times or until no debris remained on the soil surface. The areas under the tree crowns were subsequently cleared manually and all the plant remains, fallen leaves, and fruit were removed with hand rakes. The weed characteristics under the crown were similar to those between the rows of trees since the crown diameter was small and gave negligible canopy shade.

**Figure 1.**
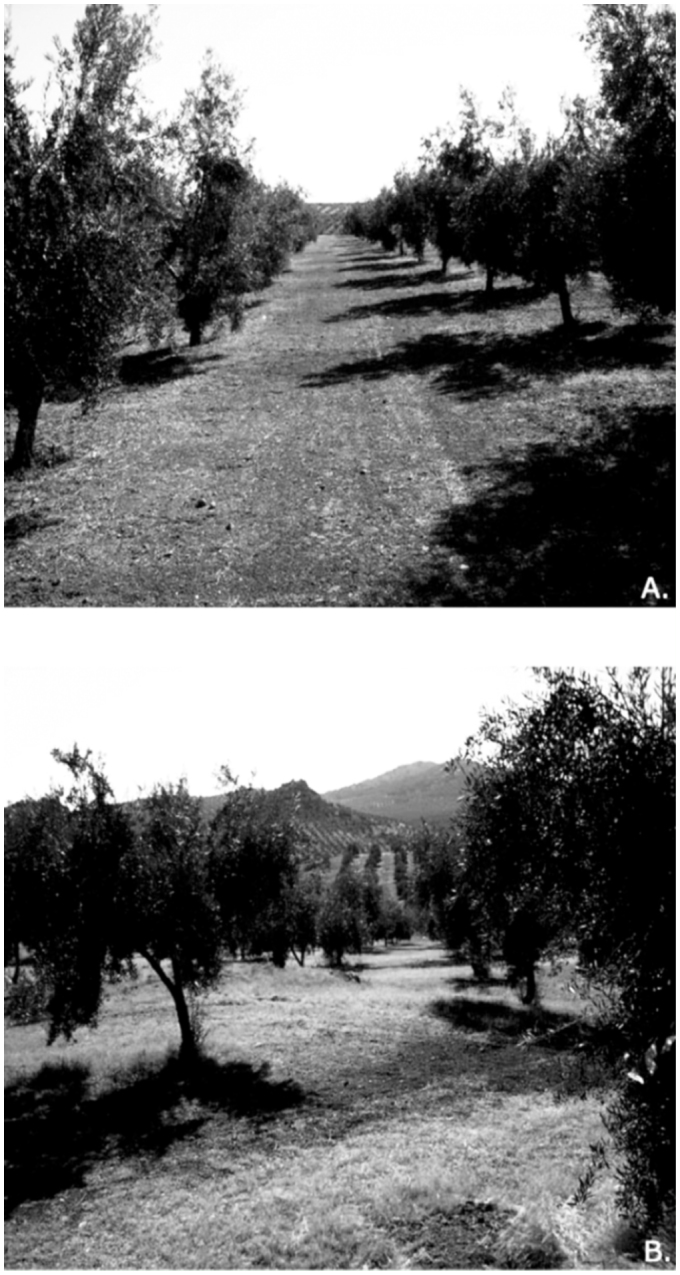
(A) View of uncovered plots (UP) during season sampling. (B) View of covered plots (CP) during season sampling. High quality figures are available online.

Three experimental uncovered plots (UPs) were chosen in which CCR was carried out as described above during the first week of June 2005 (Figure 1A). In each plot, four rows each with five trees were randomly sampled (20 trees sampled in each plot), for a total of 11 × 11 olive trees in each frame plot sampled (121 trees; 0.77 ha). There was a minimum space of 30 m between plots. In each plot, five trees were sampled, leaving one unsampled tree between analyzed trees.

A north-facing pitfall trap measuring 11 cm in diameter was placed and remained active for 48 hours. For the preservation of the individuals captured, the traps were filled with Scheerpeltz liquid (60% ethanol 97°, 38% distilled water, 1% pure acetic acid, 1% glycerine). Pitfall traps, a relatively simple capture method for collecting active arthropods on the soil surface, particularly cryptic and nocturnal ones, constitute a standard method for establishing the relative abundance of soil arthropods (Luff 1987). However, this is not a good capture method for web-building spiders in general. The fact that a large majority of spiders recorded on the olive-orchard soil were hunters was taken into account when the capture method was chosen in order to reduce the error in ecological studies of this type (Uetz and Unzicker 1976).

This design was repeated in the same orchard in three control covered plots (CPs) where the cover was not cleared (Figure 1B). In these, four rows each with five trees were sampled, for a total of 20 sampled trees per plot.

The sample season was selected according to the phenology of the cover vegetation. Four samplings were conducted in the year 2005: in the first sampling, the traps were collected 14 days after the disturbance during the last week of June; in the second sampling, 30 days after the disturbance, in the middle of July; in the third sampling, 70 days after the disturbance, in the middle of September; and in the last sampling, 85 days after the disturbance, at the end of September. The first two samplings were made to observe the short-term effects of CCR. The September samples provided data on the time course of species abundance and diversity.

All the study plots were surrounded by olive orchards under organic management, spontaneous grasses, and hedgerows with spontaneous vegetation.

### Statistical analysis

Samples were transported to the laboratory where the spiders were frozen and subsequently cleaned and identified to the lowest taxonomical level possible. The individuals identified were adults and juveniles. After identification, statistical analysis was carried out using ANOVA nonparametric statistical tests (Kruskall-Wallis test) for independent samples using the SPSS program version 13.0.

Species richness and diversity (closely correlated indices) were recorded because they were referred to as more representative diversity measures for this group (Hill 1973).

Spider diversity (*H* ^'^) was measured over the season using the Shannon-Wiener information index (Shannon 1948):


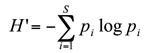


where *H* ^'^ is the amount of observed diversity in a community, *S* is the number of species (species richness), and pi is the relative abundance of the *i* ^th^ species. The greater the number of species (*S*) and the more even their abundance, the higher the diversity index *H* ^'^ will be. The maximum possible diversity for a community can be calculated as follows:


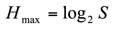


Using the equitability index (Pielou 1966), the evenness of spider species representation in the organic olive orchards was calculated as:


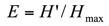


where *H* ' is the observed diversity as defined in first equation, *H*_max_ is the maximal possible diversity as defined in second equation, and *E* is the measurement of equitability. The equitability index ranges from 0 to 1, and the closer *E* is to 1, the more equally abundant are the species in the community under study.

Simpson's Index (Simpson 1949) measures “evenness” of the community from 0 to 1. Dominance (1—Simpson index) ranges from 0 (all taxa are equally present) to 1 (one taxon dominates the community completely).


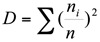


where *n*_i_ is the number of individuals of taxon *i*.

The Berger-Parker index was used to estimate dominance (Berger and Parker 1970):


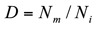


where *D* is a measure of the dominance of the most abundant species in the community (range 0 to 1), *N*_m_ is the absolute abundance of the most common species in the community under consideration, and *N*_i_ is the overall density of all the species in that community. The higher the proportion of single species, the more unevenly distributed is the species in a community.

Menhincik's richness index (Menhinick 1964) estimates the ratio of the number of taxa to the square root of the sample size:


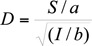


where *D* is the diversity, *S* is the number of species in whole distribution, *a* is a parameter by which the number of species is decreased with a reduced sample size, *I* is number of individuals in the whole distribution and *b* is a parameter expressing the reduction of sample size.

Margalef's richness index:


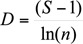


where *S* is the number of taxa and n is the number of individuals.

Equitability is Shannon diversity divided by the logarithm of number of taxa. This measures the evenness with which individuals are divided among the taxa present.

Fisher's alpha (Fisher et al. 1943) is a diversity index, defined by the formula


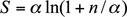


where *S* is number of taxa, *n* is number of individuals and α is the Fisher's alpha.

For the diversity study, the diversity indices were calculated using the computer programs Ecosim 7.72 (Gotelli and Enstminger 2006), divers 1.0 (Pérez-López and Sola-Fernández 1993), and PAST version 1.38 (Hammer et al. 2006). Non-parametric statistical tests (Kruskall-Wallis test) were applied to these indices to verify the possible differences between handlings and dates.

Cluster analysis allowed classified observations according to disturbance dates. The Jaccard index was used because it provided better information in terms of classification than other matching indices (Finch 2005).

## Results

### Abundance

A total of 2082 specimens were collected in the pitfall traps placed in all the sampling cycles, representing 15 families and 41 species. Of all the families captured, four families (Zodariidae, Gnaphosidae, Lycosidae, and Sicariidae) represented almost 98% of the collected specimens. The most abundant was Zodariidae, which represented more than 82% of the individuals; Gnaphosidae and Lycosidae represented 11.2 and 2.9% of the captures, respectively; and Sicariidae represented 1.1% (Table 1). For the different species, the most abundant was the only species representative of the family Zodariidae, *Zodarion styliferum* (Simon). The next species in order of abundance were two gnaphosids, *Zelotes* spp. and *Zelotes tenuis* (L. Koch) and one lycosid *Lycosa ambigua* (Barrientos), but in much lower abundance (Table 2).

**Table 1.**
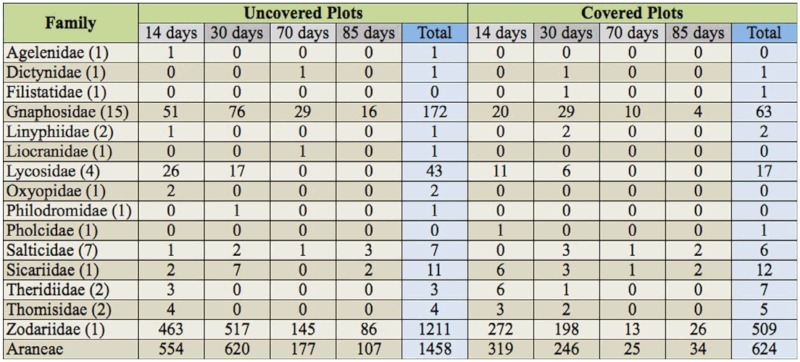
Number of specimens collected in the different families over the sampling dates (in alphabetical order), (n) Number of species collected per family.

**Figure 2.**
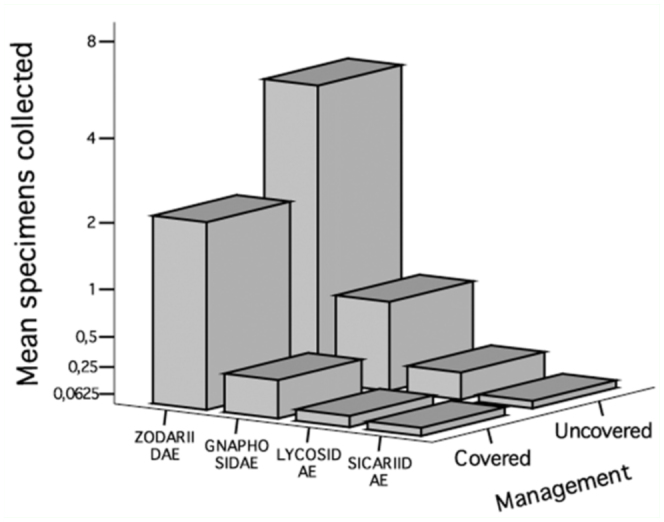
Mean number of individuals from the four main families collected in the covered and uncovered plots. High quality figures are available online.

70% of spiders were captured in UPs, while only 30% were captured in CPs. This pattern was the same among the most abundant families, such as Zodariidae, from which 70.4% of the individuals were collected from UPs. Something similar occurred with Gnaphosidae and Lycosidae, with 73.2% and 71.7% of individuals captured in UPs, respectively. However, in Sicariidae, the greatest number of individuals was found in CPs (52.2%, Figure 2). This resulted in significant differences in the total number of spiders between UPs and CPs (*p* < 0.01). For the different families, Zodariidae, Gnaphosidae, and Lycosidae presented significant differences with a greater abundance in UPs in relation to CPs (all *p* < 0.01). Sicariidae registered no significant differences in the number of individuals collected between UPs and CPs (*p* > 0.05).

**Table 2.**
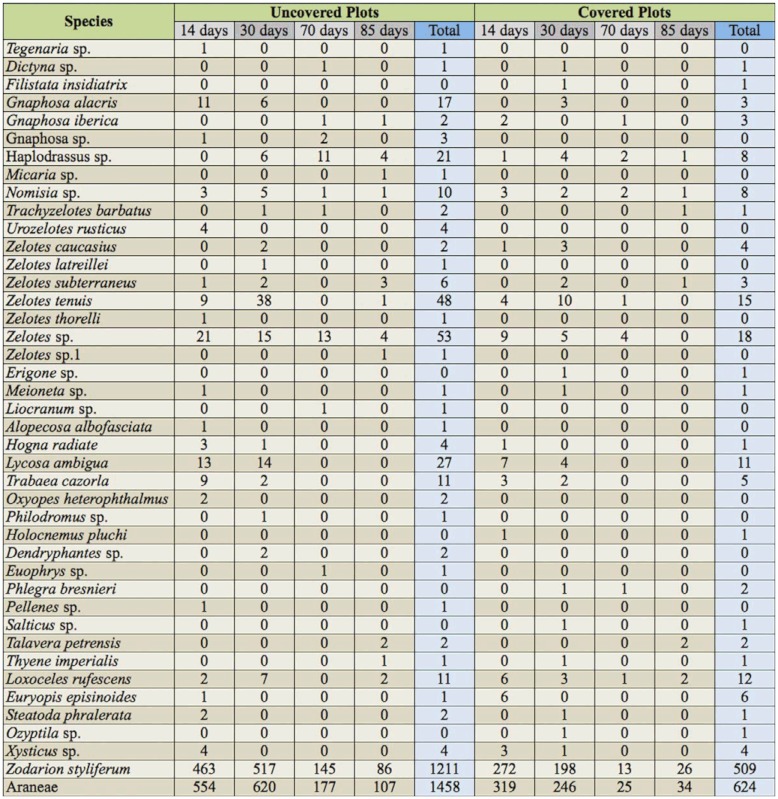
Total number of different species captured in the two treatments during the sampling cycles (species are grouped according to family).

**Figure 3.**
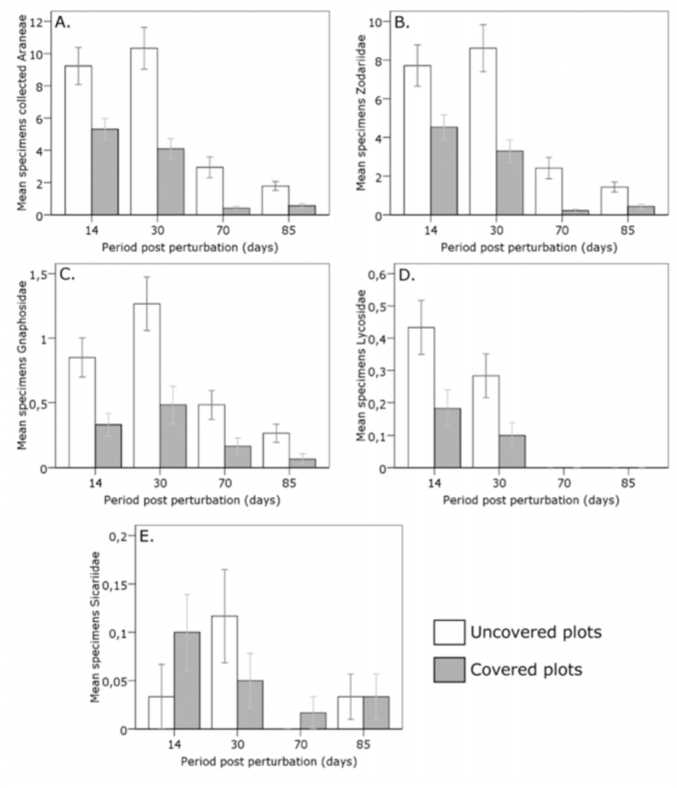
Average number of individuals collected during sampling (error bars represent ± SE). (A) Spiders (Araneae), (B) Family Zodariidae, (C) Gnaphosidae, (D) Lycosidae, (E) Sicariidae. High quality figures are available online.

In each sampling, differences were noted for the number of individuals collected and also for the four main families between the different plots (Figures 3A-E). At 14 days after CCR, CPs and UPs did not significantly differ (*p* > 0.05), whereas at 30 days, significant differences were found between UPs and CPs for the total number of spiders and also for the families Zodariidae and Gnaphosidae (all *p* < 0.01). After 70 days, significant differences were found only for the family Zodariidae, and also for the number of spiders collected (both *p* < 0.01). Finally, after 85 days, the previous case was repeated, with significant differences for the family Zodariidae and for the total number of spiders (both *p* < 0.01).

**Figure 4.**
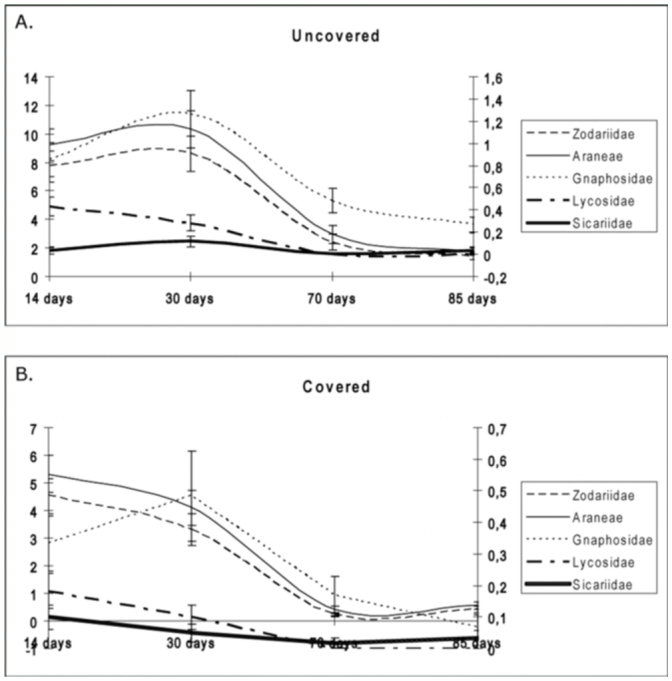
Time course of the mean number of captured spiders of the most abundant families in the study in the (A) uncovered plots and (B) covered plots. The left Y-axis represents the mean specimens of the Araneae order and Zodariidae family; right Yaxis represents the mean specimens of other families: Gnaphosidae, Lycosidae, and Sicariidae. The X-axis represents the time after CCR. High quality figures are available online.

Throughout the sampling season, a significantly greater number of spiders appeared in the first two samplings (end of June, mid-July) compared to the final two samplings (mid-September and end of September) (*p* < 0.01). In the UPs and CPs, differences were observed for the dates, with more individuals collected in the first two samplings than in the last two samplings (both *p* < 0.01, Figure 4). The most abundant family (Zodariidae) displayed significant differences between the first two and the last two samplings (*p* < 0.01). This same trend was displayed in the Gnaphosidae and Lycosidae families with declining abundance, respectively. In both cases, the differences in the number of captures between the first two and last two samplings were significant (both *p* < 0.01 respectively).

**Table 3.**
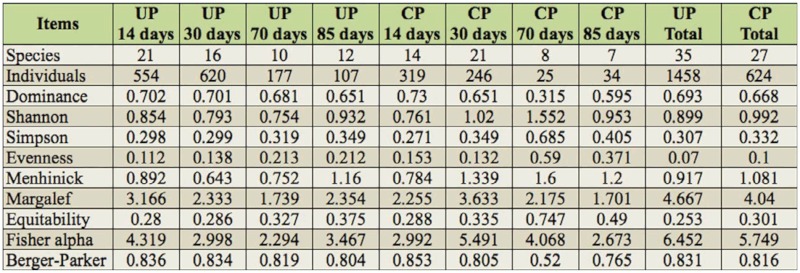
Species abundance and diversity values calculated for the different plots studied, by date and cover handling (UP uncovered plots; CP covered plots).

**Table 4.**
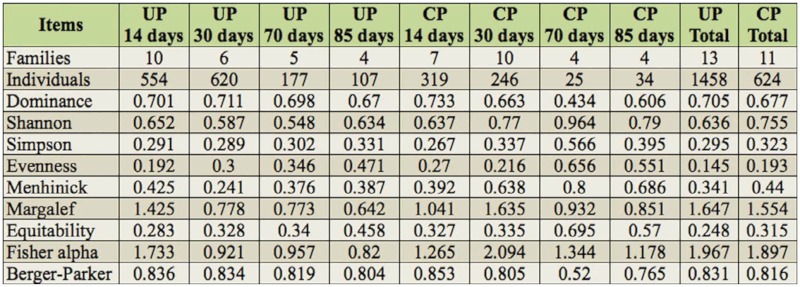
Family abundance and diversity values calculated for the different plots studied, by date and presence (C) or (U).

**Figure 5.**
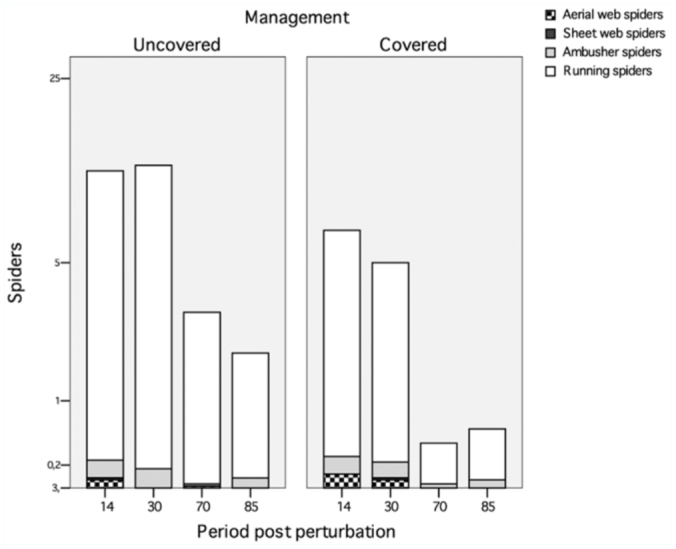
Mean number of captured spiders according to their hunting strategy. High quality figures are available online.

The majority of the captured species belonged to the guild of cursorial spiders (families Zodariidae, Gnaphosidae, Liocranidae, and Lycosidae), followed by ambusher spiders (families Oxyopidae, Philodromidae, Salticidae, Sicariidae, and Thomisidae), and with little representation of web-building spiders, whether sheet-web builders (Agelenidae and Filistatidae) or aerial-web builders (Families Dictynidae, Linyphiidae, Pholcidae, Theridiidae; Figure 5). Although there were no differences in the distribution of the captures between these groups of spiders, running spiders significantly differed between UPs and CPs (*p* < 0.01). No differences were found for the other groups of spiders collected (ambushers and sheet-web or aerial-web builders, *p* > 0.05 in all cases).

## Diversity

**Figure 6.**
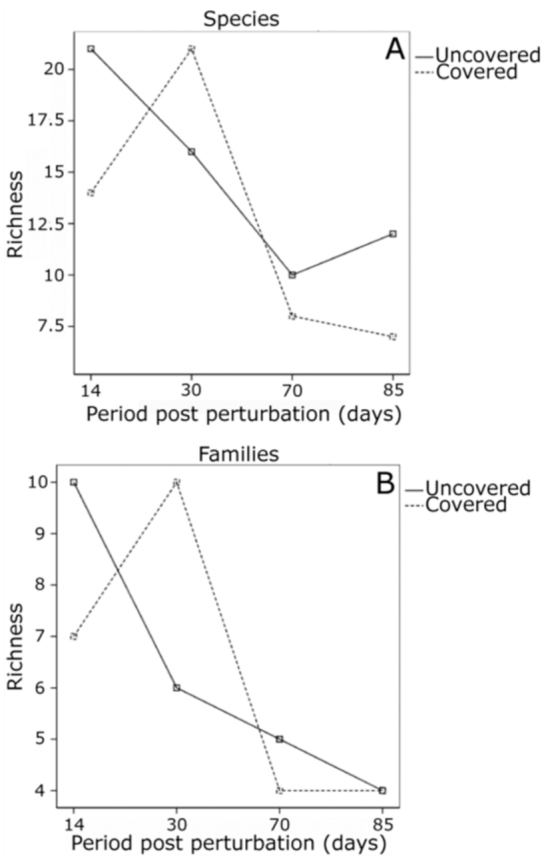
Time course of diversity measured in terms of richness over the sampling season. (A) Species richness. (B) Time course of diversity measured in terms of family richness over the sampling season. High quality figures are available online.

The diversity results (Tables 3 and 4) gave a total of 35 species belonging to 13 families in UPs and 27 species of 11 families in CPs. Significant differences appeared between CPs and UPs for species richness, this being greater in UPs (*p* < 0.05). Throughout the year, a greater number of species were observed in the first two samplings (those of June and July) and a subsequent decrease in the last two (Figure 6A). Similarly, the number of families collected declined over the sampling season (Figure 6B).

The diversity indices on the different sampling dates did not show any significant differences for any of the indices calculated. Only in the case of Shannon's diversity index were any significant differences recorded between the families collected in UPs and those collected in CPs (*p* < 0.05) across all sample dates.

For all the families except Zodariidae, significant differences were recorded for Hulbert's diversity index between UPs and CPs (Chi-squared = 6.277; df = 1;*p* < 0.05).

In terms of dominance, no significant differences (*p* > 0.05) were detected between plots on different sampling dates. However, significant differences were found for dominance (which was greater in CPs) when the family Zodariidae (*p* < 0.05) was excluded from the analysis.

**Figure 7.**
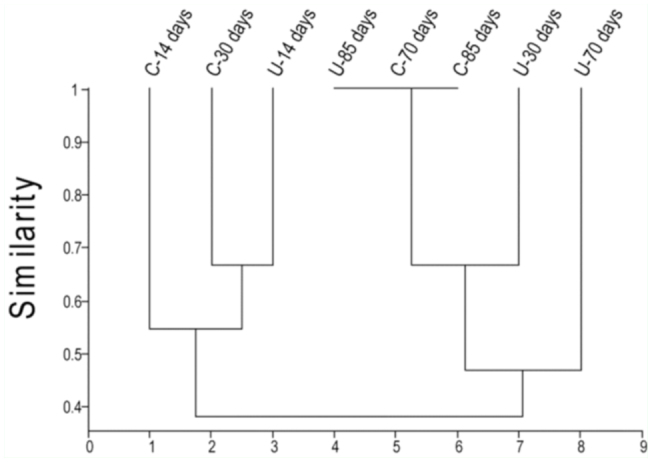
Dendrogram resulting from the multivariate analysis of paired groups calculated for the Jaccard index. Correlation coefficient 0.755. High quality figures are available online.

**Figure 8.**
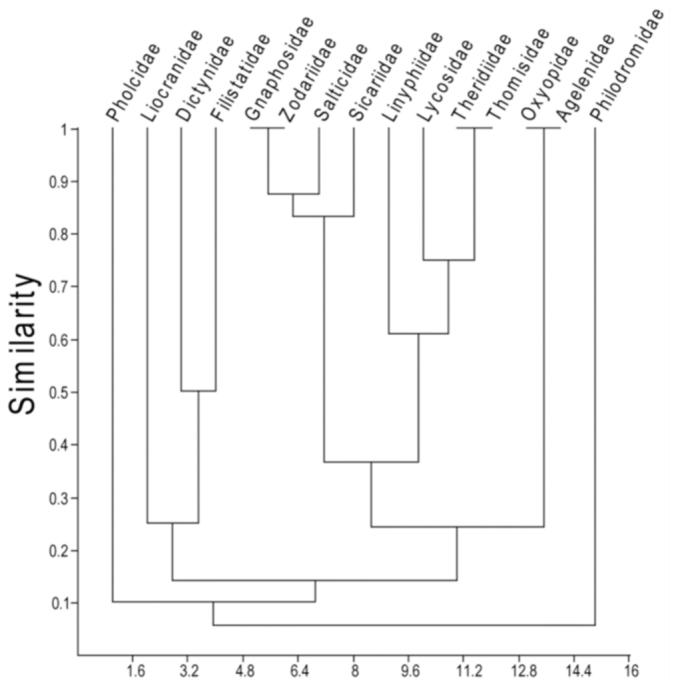
Dendrogram resulting from the multivariate analysis of paired groups calculated for the Jaccard index. Correlation coefficient 0.9654. High quality figures are available online.

When a cluster analysis was performed, there were differences only for the time but not for the management (Figure 7). Additionally, there were no differences recorded in the distribution of families between UPs and CPs, since the most abundant family (Zodariidae) showed little similarity with the other families in terms of the distribution between treatments over time (Figure 8).

## Discussion

### Faunistic composition

In the present study, the taxonomical composition of spiders for the most abundant taxa was limited to families of cursorial spiders: Zodariidae, Gnaphosidae, Lycosidae, and Sicariidae. It also included other grounddwelling specialist spiders belonging to the families Theridiidae, such as *Steatoda phralerata* (Panzer) and *Euryopis episinoides* (Walckenaer) (also ant-eaters, as zodariids), and Thomisidae (*Xysticus* genus).

**Figure 9.**
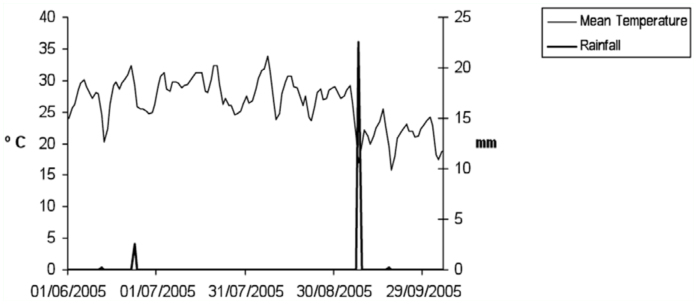
Temperature (^°^C) and rainfall (mm) during sampling from Mancha Real meteorological station (Weather stations, Consejería de Agricultura y Pesca, Junta de Andalucía, 2006). High quality figures are available online.

In view of the sampling results, the taxonomical composition of spiders was strongly influenced by at least two factors:

(1) The small size of this year's cover crop, due to the shortage of rain and high temperatures during samplings (Figure 9). This presumably prevented greater differences from being recorded, and therefore families normally collected in olive orchards with low cover crops, as for example the family Linyphiidae, were not collected here in large numbers due to the vegetation not having the sufficient size or structure necessary for the placement of the laminar capture webs. Another example would be provided by the spiders of the genus *Oxyopes* (family Oxyopidae), which also inhabit this stratum of scant vegetation and are abundant in olive orchards with crop cover (Cárdenas pers. obs.).

(2) In addition, when the disturbance occurs (cover crop removal), this results in colonizing species moving into this “empty” area from surrounding patches with crop cover, both from other covered plots and also from other bordering vegetation patches. Therefore, in this experiment, species of Gnaphosidae (e.g., various species of the genus *Zelotes*) were captured in similar numbers in the two types of plots, since errant spiders colonize the free space. In the case of species of the family Lycosidae, such as *Hogna radiata* (Latreille) and *Lycosa ambigua*, one explanation could be the territoriality exhibited by species of this family. Specimens without hunting territories seek to increase prey availability (Moya-Laraño et al. 2002) in these disturbed but as of yet unoccupied plots.

### Abundance

The greater abundance found in plots where the crop cover was removed might be due to the greater capacity of species of cursorial spider families (families Zodariidae, Lycosidae, and Gnaphosidae) to occupy the space created after the disturbance by CCR. However, it should be emphasised that highly mobile species will be more frequently and abundantly collected in a pit-fall trap due to their behavior. Since Zodariidae frequently construct silken retreats in ground debris and/or below ground (Jocqué 1991; Ramirez 1995), they found better shelter options in uncovered plots that offer a clear environment to place their shelter under stones as other *Zodarion* species (Pekár 2004). In fact, the uncovered soil offers a lower resistance to spider dispersal compared to a covered soil, causing an apparent increase in spider abundance. In the case of the family Zodariidae, these spiders—specialized anteaters (Jocqué 1991)—could continue the displacement criteria of their prey, some ant species, who take advantage of the space created by the disturbance caused by cover crop removal to colonize this free space and to build their nests. Habitat structure is fundamental for ant dispersal strategies (Heinze 1993). Ants modify their pattern of exploitation of the area after the disturbance. The same presumably occurred in our study as in other cases of disturbances (e.g., fire) in that abundance first increased, but then decreased progressively to reach values closer to those of the undisturbed area (Garcia et al. 1995). Furthermore, *Zodarion* reportedly prey more easily on ant species of the subfamily Formicinae (such as *Cataglyphis* and *Camponotus)* than on species of the subfamily Myrmicinae (such as *Messor* or *Tetramorium)* (Pekár 2004). The ant-abundance results reveal greater abundance of individuals in the subfamily Formicinae (with more than 50% of the captures) in UPs than in CPs, where values represented only 31% and Myrmicinae predominated, explaining the greater abundance of these spiders in UPs (Campos et al. 2011). This appears to be due to the fact that ants of the subfamily Formicinae are paralyzed more quickly by the venom of spiders of the genus *Zodarion* than that of the subfamily Myrmicinae (Pekár 2005).

Few differences were found between CPs and UPs, perhaps because of the very sparse cover in CPs, together with a low impact of the different agricultural practices. In summary, pitfall traps show insect activity that can be higher under specific conditions (i.e. less structured vegetation).

The families Lycosidae and Gnaphosidae showed a trend similar to that of the family Zodariidae, with greater abundance in plots without a cover crop. Despite these differences, the time course of the captures in both plots proved similar (Figure 3). In both families, the disturbance (i.e., CCR) would cause a migration from areas with cover crops, less prey, greater competition, other predators, and other spiders (with other hunting strategies), towards uncovered areas with more prey and less competition. Furthermore, observation of the lycosids revealed that they did not vary their phenological cycle, registering a greater abundance in the months of June and July, and declining in September. In the last sampling, no individuals of this family were collected in any of the plots.

### Diversity

Species richness significantly increased in uncovered plots because, like other organisms (plants), disturbance temporarily elevated the importance of spontaneous/pioneer species, which were quickly replaced by other species better adapted over time (De Cauwer et al. 2005; Cauwer et al. 2006). This affects herbivorous insect populations in different ways (Pöyry et al. 2006) and thus affects predators (Hendrikx et al. 2007).

The diversity results do not indicate any differences except in the case of Shannon's diversity index calculated for the families, which is one of the most appropriate diversity indices for litter-diversity measure in arthropods (Leponce et al. 2004). A greater number of families in UPs would reflect the fact that, of these, the greatest number belongs to spider families, the main characteristic of which is a large dispersal capacity either because of their hunting strategy as active cursorial spiders (families such as Zodariidae, Gnaphosidae, Lycosidae, Sicariidae), or because of their high dispersal capacity (e.g., families Salticidae or Linyphiidae). Differences in dispersal capabilities also occur with other arthropods, as in the case of coleopterans, in which the taxa with a higher dispersal capacity (greater flying capacity) are the ones that first colonize rural and urban areas that have been disturbed by humans (Venn et al. 2003).

Two taxonomic levels were used for the diversity results according to the two most widely used techniques: the taxonomic surrogacy for a rapid assessment of biodiversity in agroecosystems (Biaggini et al. 2007) and the trough species level (Bertrand et al. 2006).

When the dominant species in Z. *styliferum* was removed, the diversity results failed to reveal any significant differences except for the diversity index of Hulbert's PIE between CPs and UPs, with a greater diversity in UPs, probably due to the taxonomical composition of mainly spiders which actively move from one place to another.

The results agreed with the fact that temporal variations in species abundance also affects the measure of diversity (Leponce et al. 2004). Time was found to be the most important factor to explain spider species presence in olive orchards.

Finally, the results of the similarity cluster analysis revealed no clear grouping to indicate a trend concerning the effects of an intermediate disturbance on species distribution in the olive orchard (Figure 8). Additionally, the families did not show any pattern of distribution in accordance with the degree of disturbance between UPs and CPs (Figure 7), although this might be due to the narrow time range studied.

### Conclusions

These results indicate that a disturbance such as CCR in an olive orchard has a positive effect on the abundance of certain spider species, such as Z. *styliferum* and other running spiders of the families Gnaphosidae and Lycosidae. Throughout the postdisturbance period, the differences found between the CPs and the UPs were even more pronounced in families such as Zodariidae than in other running spiders.

However, cover removal does not have a strongly significant effect on diversity. The greater diversity found in the UPs might be due to disturbance (i.e., CCR), which means that taxa present in adjacent areas take advantage of the newly created ecological niche to colonize it.

There is one finding that slightly complicates the conclusions and that could mask certain results—the large number of Z. *styliferum* individuals. In future samplings, it would be useful to determine whether their high number responds to the direct effect of the disturbance or is due to their normal population dynamics in this region. Further studies are recommended at greater temporal and spatial scales in order to verify long-term effects of an intermediate disturbance (e.g., CCR) on the abundance and diversity of soil spiders in olive orchards.

## **Abbreviations**

CCR: cover-crop removal;
CP: covered plot;
UP: uncovered plot
